# Effects of Wolf Mortality on Livestock Depredations

**DOI:** 10.1371/journal.pone.0113505

**Published:** 2014-12-03

**Authors:** Robert B. Wielgus, Kaylie A. Peebles

**Affiliations:** Large Carnivore Conservation Laboratory, School of Environment, Washington State University, Pullman, Washington, United States of America; Michigan Technological University, United States of America

## Abstract

Predator control and sport hunting are often used to reduce predator populations and livestock depredations, – but the efficacy of lethal control has rarely been tested. We assessed the effects of wolf mortality on reducing livestock depredations in Idaho, Montana and Wyoming from 1987–2012 using a 25 year time series. The number of livestock depredated, livestock populations, wolf population estimates, number of breeding pairs, and wolves killed were calculated for the wolf-occupied area of each state for each year. The data were then analyzed using a negative binomial generalized linear model to test for the expected negative relationship between the number of livestock depredated in the current year and the number of wolves controlled the previous year. We found that the number of livestock depredated was positively associated with the number of livestock and the number of breeding pairs. However, we also found that the number of livestock depredated the following year was positively, not negatively, associated with the number of wolves killed the previous year. The odds of livestock depredations increased 4% for sheep and 5–6% for cattle with increased wolf control - up until wolf mortality exceeded the mean intrinsic growth rate of wolves at 25%. Possible reasons for the increased livestock depredations at ≤25% mortality may be compensatory increased breeding pairs and numbers of wolves following increased mortality. After mortality exceeded 25%, the total number of breeding pairs, wolves, and livestock depredations declined. However, mortality rates exceeding 25% are unsustainable over the long term. Lethal control of individual depredating wolves may sometimes necessary to stop depredations in the near-term, but we recommend that non-lethal alternatives also be considered.

## Introduction

Predator control and sport hunting are often used to reduce predator populations, livestock depredations, and increase social acceptance of large carnivores such as brown bears (*Ursus arctos*) [1], wolves (*Canis lupus*) [2], cougars (*Puma concolor*) [3], jaguars (*Panthera onca*) [4], lions (*Panthera leo*) [5], leopards (*Pantera pardus*) [6], and others [7].

Gray wolves (our model animal) are currently being hunted in Idaho, Wyoming and Montana, in part, to reduce livestock depredations [8]. However, to our knowledge, the long-term effectiveness of lethal wolf control to reduce livestock depredations has not yet been rigorously tested. For example, Bradley and Pletscher [9] predicted that breeding pairs are responsible for most livestock depredations because they are bound to the den site, not natural prey distribution [10]. Brainerd et al. [11] predicted that increased wolf mortality could result in fracture of pack structure and increased breeding pairs. If this is the case, increased mortality of wolves could result in increased breeding pairs and livestock depredations following lethal control. In other species, Collins et al. [12] and Treves et al. [13] found increased damages by black bears (*Ursus americanus*) following high remedial mortality and Peebles et al. [14] found that increased mortality of cougars resulted in increased livestock depredations because of social disruption. In this paper we test the widely accepted, but untested, hypothesis that increased lethal control decreases wolf livestock depredations in a large scale (tri-state) long-term (25 year) quasi-experimental [15]. The “remedial control” hypothesis predicts that livestock depredations will decrease following increased lethal control.

## Methods

We obtained the confirmed number of cattle (*Bos primigenius*) and sheep (*Ovis aries*) depredated, wolf population estimates, number of breeding pairs, and the number of wolves killed in the wolf-occupied area of each state for each year between 1987–2012 from United States Fish and Wildlife Services Interagency Annual Wolf Reports [16] (Table S1). The number of wolves killed were wolves that were killed through control methods including wolves killed legally by livestock owners or through government control methods, these numbers do not include other sources, including natural mortality. Only the total numbers of livestock killed, not the number of confirmed livestock depredation incidents, were available from the USFW database.

Numbers of livestock were similarly obtained from United States Department of Agriculture National Agricultural Statistics Service for counties where wolves are present [17] (Table S1). Livestock numbers for individual properties were not available so livestock tallies were made across the tri-state area using counts from wolf occupied counties.

Following Peebles et al. [14], we used forward selection, negative binomial general linearized models to assess the relationship between livestock depredations and numbers of livestock, wolves, breeding pairs, and wolves killed. We used this method because depredations were over-dispersed and consisted of 0 to positive integer count data with a variance exceeding the mean [18]. The best statistical model was then selected using the lowest AIC (Akaike Information Criterion) and highest log-likelihood [19]. The rate ratio, analogous to odds-ratio, was computed from the coefficients to aid in interpreting the results [20]. For example, a rate ratio of 1.0 for any independent variable means the effect on the dependent variable is unchanged. A rate ratio of 1.5 means the odds are increased by 50%.

To establish directionality, we analyzed the effect of independent variables in year 1 on number of livestock depredated in year 2. This one year time-lag between control kills and depredations removes the directional effect of depredations causing kills. After assessing the models, we plotted and interpreted the most important independent variables against depredations to provide a visual representation of model terms. Only the independent variables that had a rate ratio larger than 1.01or smaller than 0.99, meaning the change in the mean number of livestock depredated was increased or decreased by at least 1%, were examined further. We conducted our regressions on the entire tri-state area and the 3 separate states- but only report the larger tri-state area here because the results were basically the same in all cases (Figures S1 and S2).

## Results

The total number of livestock depredated between January 1987- December 2012 in the tri-state area was 5670; 1853 were cattle and 3723 were sheep. Sample size for paired depredations in year 2 and wolf and cattle numbers and wolves killed in year 1 was: 17 years for Idaho, 17 years for Wyoming, and 25 years for Montana (N total  = 59).

All of the well performing models (AIC<466) for cattle depredated included the # wolves killed, # of breeding pairs and the # of wolves killed by # breeding pairs interaction (Table 1) - and the coefficients for these terms were very similar across models. The best models were #10, #12 and #13. The 1^st^ model was g(y)  =  exp [1.307+0.05078(wolves killed through control methods) +0.07979(# of breeding pairs) +2.116×10^−8^(# of cattle) – 1.343×10^−3^(# breeding pairs*wolves killed) – 2.980×10^−8^(# of cattle*wolves killed)]. The 2^nd^ model was g(y)  =  exp [1.142+0.05293(wolves killed through control methods) +0.08791(# of breeding pairs) +2.674×10^−7^(# of cattle) – 0.001377(wolves killed*# of breeding pairs) – 1.701×10^−8^(# of cattle*# of breeding pairs)]. The 3^rd^ model was g(y)  =  exp [1.182+0.05795(wolves killed through control methods) +0.07783(# of breeding pairs) +2.112×10^−7^(# of cattle) – 0.001378(wolves killed*# of breeding pairs) – 7.804×10^−9^(# of cattle*# of wolves killed)]

**Table 1:** AIC and log-likelihood values for forward selection of main effects and interaction effects models of cattle depredations

Model #	Main Effects	Interaction Effects	AIC	2× log-likelihood
1	Cattle	N/A	528.19	−522.188
2	# of breeding pairs	N/A	486.13	−480.132
3	wolves killed	N/A	494.11	−488.111
4	cattle + breeding pairs	N/A	488.08	−480.079
5	cattle + wolves killed	N/A	489.86	−481.857
6	breeding pairs + wolves killed	N/A	484.61	−476.610
7	cattle + breeding pairs + wolves killed	N/A	485.40	−475.397
8	cattle + breeding pairs + wolves killed	cattle * breeding pairs	487.36	−475.360
9	cattle + breeding pairs + wolves killed	cattle * wolves killed	487.34	−475.336
10	cattle + breeding pairs + wolves killed	breeding pairs * wolves killed	464.02	−452.018
11	cattle + breeding pairs + wolves killed	cattle * breeding pairs; cattle * wolves killed	488.8	−474.803
12	cattle + breeding pairs + wolves killed	cattle * breeding pairs; breeding pairs * wolves killed	465.67	−451.668
13	cattle + breeding pairs + wolves killed	cattle * wolves killed; breeding pairs * wolves killed	465.39	−451.392
14	cattle + breeding pairs + wolves killed	cattle * breeding pairs; cattle * wolves killed; wolves killed * breeding pairs	467.36	−451.358

**Figure 1:**
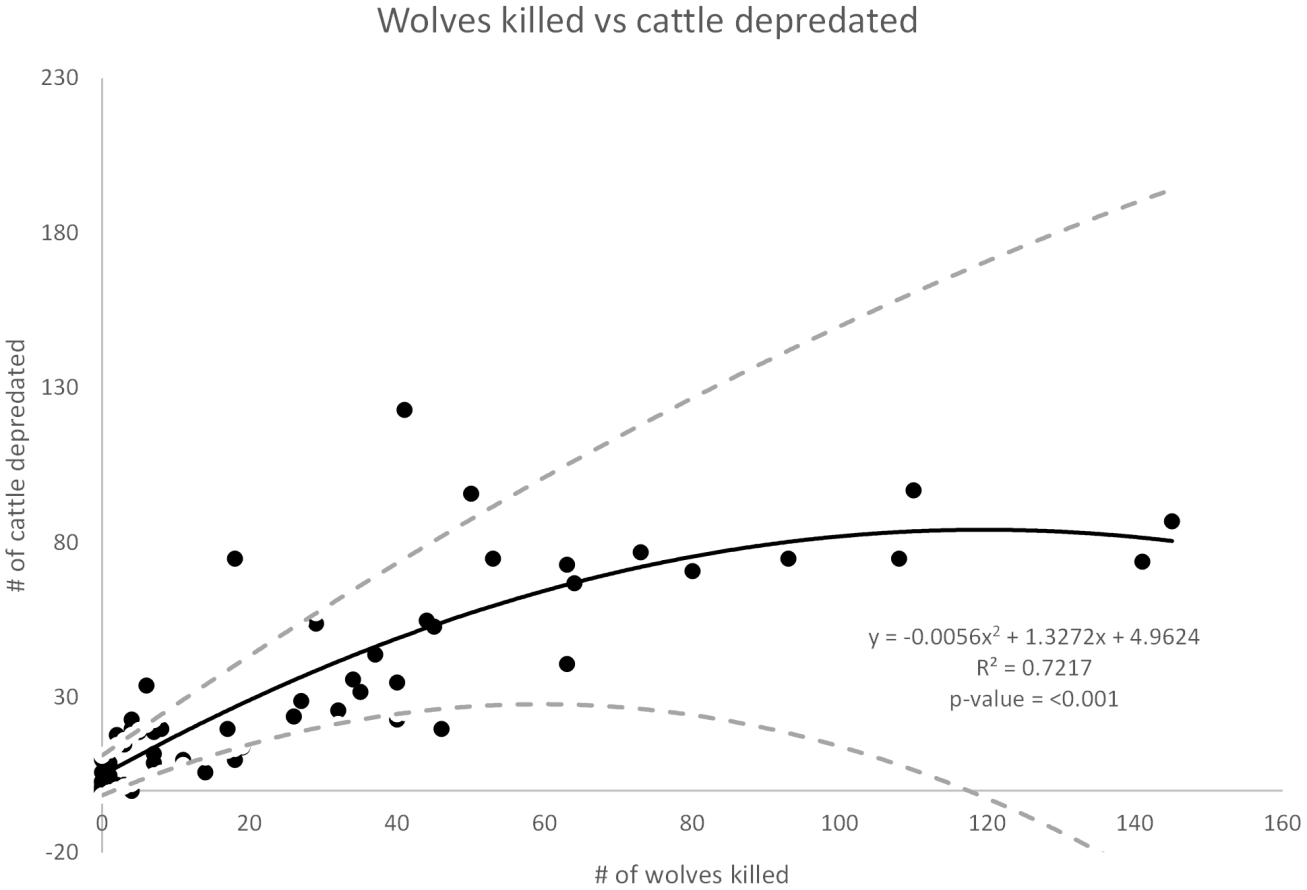
Wolves killed vs cattle depredated. Number of wolves killed through control methods the previous year versus the number of cattle depredated the following year. The dashed lines show the upper and lower limits of the 95% confidence interval for the best fit line.

**Figure 2:**
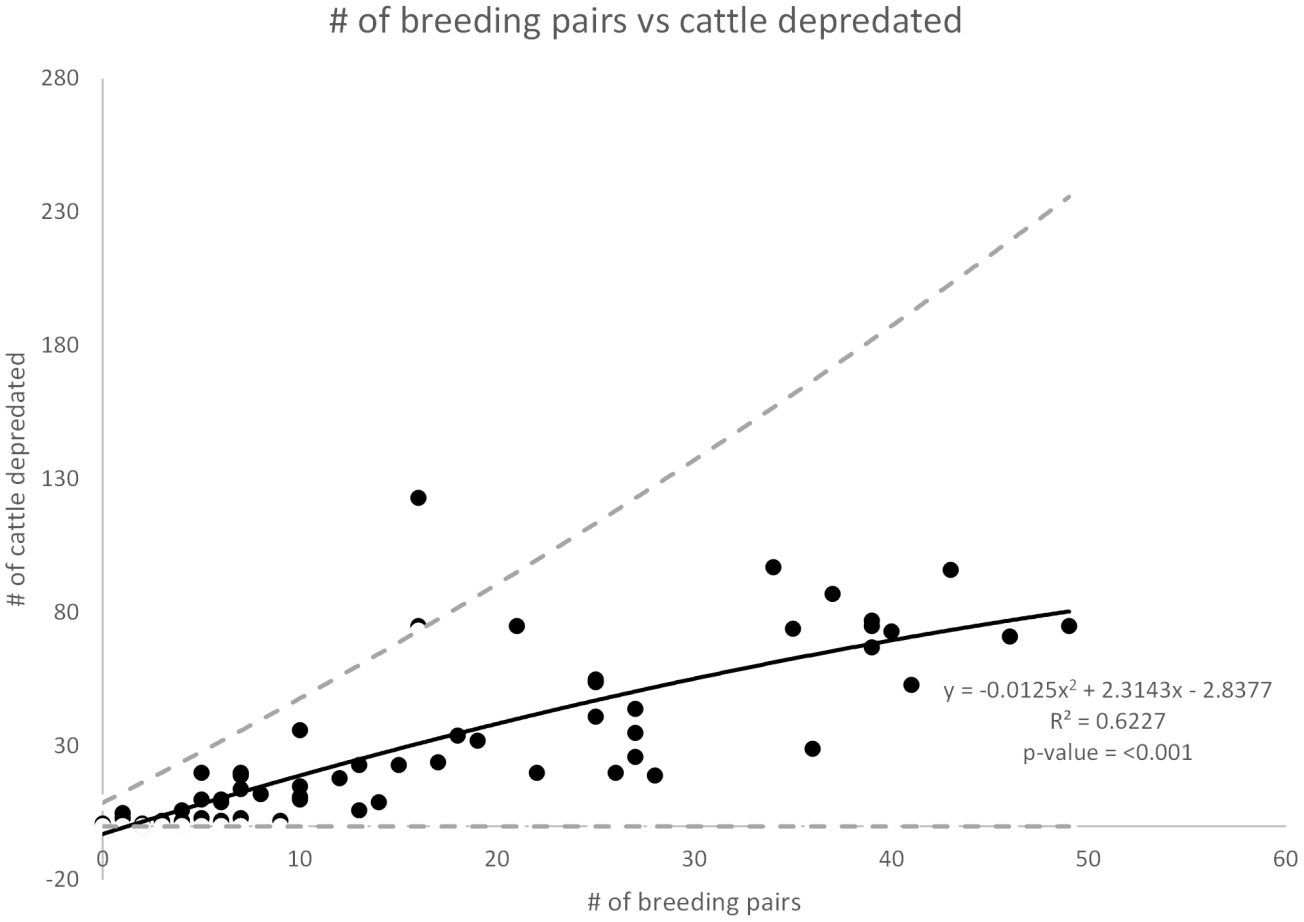
Number of breeding pairs vs cattle depredated. Number of breeding pairs present on the landscape the previous year versus the number of cattle depredated the following year. The dashed lines show the upper and lower limits of the 95% confidence interval for the best fit line.

In both models all of the main effects and some two way interactions were found to be statistically significant (Table 2). The number of wolves killed in year one was positively related to the number of cattle depredated the following year (rate ratios  = 1.05, 1.05 and 1.06, *z* = 5.67 and 5.66, 4.69, *P*<0.001) (Figure 1). For each additional wolf killed the estimated mean number of cattle depredated the following year increased by 5 to 6%. The number of breeding pairs was also positively related to the number of cattle depredated (rate ratios  = 1.08, 1.09 and 1.08, *z* = 6.28, 4.87 and 6.04, *P* = 0.0336 and <0.001) (Figure 2). For each additional breeding pair on the landscape the estimated mean number of cattle depredated the following year increased by 8 to 9%. Breeding pairs were highly correlated with numbers of wolves (Table S2).

**Table 2:** Summary of best model for cattle depredated.

Dependent Variable	Independent Variable	Estimated Coefficients	Standard Error	VIF (variance inflation factor)	Z value	p-value	AIC
**Cattle Depredated**	Cattle	2.116×10^−8^	3.701×10^−7^	1.38	0.057	0.954	464.02
	# of breeding pairs	.07979	.01270	3.87	6.281	<0.001	
	# of wolves killed	.05078	8.925×10^−3^	3.41	5.689	<0.001	
	# breeding pairs*# of wolves killed	−1.343×10^−3^	2.492×10^−4^		−5.338	<0.001	
**Cattle Depredated**	Cattle	2.674×10^−7^	5.493×10^−7^		0.487	0.626	465.67
	# of breeding pairs	.08791	.01804		4.872	<0.001	
	# of wolves killed	.05293	9.354×10^−3^		5.658	<0.001	
	Cattle*# of breeding pairs	−1.701×10^−8^	2.809×10^−8^		−0.605	0.545	
	# of breeding pairs*# of wolves killed	−1.377×10^−3^	2.511×10^−4^		−5.485	<0.001	
**Cattle Depredated**	Cattle	2.112×10^−7^	4.477×10^−7^		0.472	0.637	465.39
	# of breeding pairs	.07783	.01289		6.036	<0.001	
	# of wolves killed	.05795	.01236		4.689	<0.001	
	Cattle*# of wolves killed	−7.804×10^−9^	9.865×10^−9^		−0.791	0.429	
	# of breeding pairs* # of wolves killed	−1.378×10^−3^	2.498×10^−4^		−5.515	<0.001	

**Figure 3:**
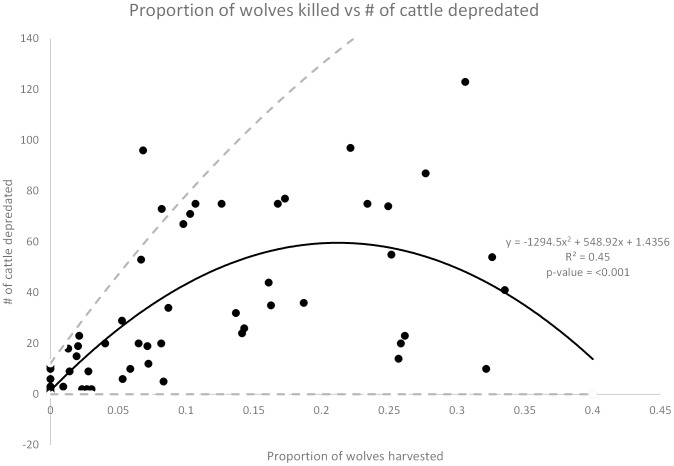
The proportion of wolves killed vs cattle depredated. Proportion of wolves killed the previous year versus the number of cattle depredated the following year. The dashed lines show the upper and lower limits of the 95% confidence interval for the best fit line.

There was also one important 2-way negative interaction for the relationship between the increasing numbers of wolves killed and decreasing breeding pairs on livestock depredations (rate ratios  = 0.99, *z* = −5.39, −5.49 and −5.12, *P*<0.001. In our models, the main effects of wolves killed was increased depredations. But the negative interaction effect in the model shows that depredations ultimately declined with increased wolf kills as number of breeding pairs decreased. These conflicting effects on livestock depredations are represented here as proportion of wolves killed vs. cattle depredations in (Figure 3). Depredations increased with increasing wolf mortality up to about 25% mortality but then depredations declined when mortality exceeded 25%.

One model out of 53 (Table 3) was also selected for determining which factors may influence the number of sheep depredated the following year (Table 4). The model was g(y)  =  exp [−10.499+0.05539(minimum wolf population) +0.03883(wolves killed through control methods) +3.058×10^−5^(cattle) +2.077×10^−4^(sheep) – 5.116×10^−4^(wolves killed*wolf population) – 4.932×10^−7^(wolves killed*cattle) – 1.159×10^−7^(wolf population*cattle) – 3.712×10^−6^(wolves killed*sheep) – 6.827×10^−7^(wolf population*sheep) – 3.408×10^−10^(cattle*sheep) +6.532×10^-10^(wolves killed*wolf population*cattle) +4.819×10^−9^(wolves killed*wolf population*sheep) +3.682×10^−12^(wolves killed*cattle*sheep) – 4.336×10^−15^(wolves killed*wolf population*cattle*sheep)].

**Table 3:** AIC and log-likelihood values for forward selection of main effects and interaction effects models of sheep depredations.

Model #	Main Effects	Interaction Effects	AIC	2× log-likelihood
1	Wolves killed	N/A	575.92	−569.925
2	Cattle	N/A	581.06	−575.058
3	Sheep	N/A	560.08	−554.077
4	Wolf population	N/A	563.61	−557.605
5	Wolves killed + cattle	N/A	573.4	−565.401
6	Wolves killed + sheep	N/A	561.86	−553.861
7	Wolves killed + wolf population	N/A	565.19	−557.192
8	Cattle + sheep	N/A	561.81	−553.809
9	Cattle + wolf population	N/A	565.20	−557.205
10	Sheep + wolf population	N/A	558.89	−550.889
11	Wolves killed + cattle + sheep	N/A	563.51	−553.513
12	Wolves killed + sheep + wolf population	N/A	560.43	−550.432
13	Cattle + sheep + wolf population	N/A	560.88	−550.882
14	Wolves killed + cattle + sheep + wolf population	N/A	562.41	−550.406
15	Wolves killed + cattle + sheep + wolf population	Wolves killed*cattle	564.33	−550.334
16	Wolves killed + cattle + sheep + wolf population	Wolves killed*sheep	553.78	−539.780
17	Wolves killed + cattle + sheep + wolf population	Wolves killed*wolf population	556.00	−542.004
18	Wolves killed + cattle + sheep + wolf population	Cattle*sheep	549.98	−535.978
19	Wolves killed + cattle + sheep + wolf population	Cattle*wolf population	563.62	−549.622
20	Wolves killed + cattle + sheep + wolf population	Sheep*wolf population	551.13	−537.127
21	Wolves killed + cattle + sheep + wolf population	Wolves killed*cattle, wolves killed*sheep	553.70	−537.698
22	Wolves killed + cattle + sheep + wolf population	Wolves killed*cattle, wolves killed*wolf population	557.64	−541.643
23	Wolves killed + cattle + sheep + wolf population	Wolves killed*cattle, cattle*sheep	548.16	−532.159
24	Wolves killed + cattle + sheep + wolf population	Wolves killed*cattle, cattle*wolf population	563.36	−547.355
25	Wolves killed + cattle + sheep + wolf population	Wolves killed*cattle, sheep*wolf population	548.38	−532.378
26	Wolves killed + cattle + sheep + wolf population	Wolves killed*sheep, wolves killed*wolf population	555.78	−539.778
27	Wolves killed + cattle + sheep + wolf population	Wolves killed*sheep, cattle*sheep	551.82	−535.819
28	Wolves killed + cattle + sheep + wolf population	Wolves killed*sheep, cattle*wolf population	554.61	−538.608
29	Wolves killed + cattle + sheep + wolf population	Wolves killed*sheep, sheep*wolf population	553.10	−537.099
30	Wolves killed + cattle + sheep + wolf population	Wolves killed*wolf population, cattle*sheep	551.38	−535.376
31	Wolves killed + cattle + sheep + wolf population	Wolves killed*wolf population, cattle*wolf population	557.98	−541.981
32	Wolves killed + cattle + sheep + wolf population	Wolves killed*wolf population, sheep*wolf population	553.12	−537.120
33	Wolves killed + cattle + sheep + wolf population	Cattle*sheep, cattle*wolf population	548.34	−532.342
34	Wolves killed + cattle + sheep + wolf population	Cattle*sheep, sheep*wolf population	551.11	−535.115
35	Wolves killed + cattle + sheep + wolf population	Cattle*wolf population, sheep*wolf population	549.75	−533.754
36	Wolves killed + cattle + sheep + wolf population	Wolves killed*cattle, wolves killed*sheep, wolves killed*wolf population	555.40	−537.401
37	Wolves killed + cattle + sheep + wolf population	Wolves killed*cattle, wolves killed*sheep, cattle*sheep	549.67	−531.675
38	Wolves killed + cattle + sheep + wolf population	Wolves killed*cattle, wolves killed*sheep, cattle*wolf population	555.39	−537.391
39	Wolves killed + cattle + sheep + wolf population	Wolves killed*cattle, wolves killed*sheep, sheep*wolf population	550.30	−532.304
40	Wolves killed + cattle + sheep + wolf population	Wolves killed*sheep, wolves killed*wolf population, cattle*sheep	553.09	−535.090
41	Wolves killed + cattle + sheep + wolf population	Wolves killed*sheep, wolves killed*wolf population, cattle*wolf population	556.36	−538.360
42	Wolves killed + cattle + sheep + wolf population	Wolves killed*sheep, wolves killed*wolf population, sheep*wolf population	555.00	−537.003
43	Wolves killed + cattle + sheep + wolf population	Wolves killed*wolf population, cattle*sheep, cattle*wolf population	549.86	−531.863
44	Wolves killed + cattle + sheep + wolf population	Wolves killed*wolf population, cattle*sheep, sheep*wolf population	553.06	−−535.059
45	Wolves killed + cattle + sheep + wolf population	Cattle*sheep, cattle*wolf population, sheep*wolf population	548.20	−530.203
46	Wolves killed + cattle + sheep + wolf population	Wolves killed*cattle, wolves killed*sheep, wolves killed*wolf population, cattle*sheep	551.60	−531.602
47	Wolves killed + cattle + sheep + wolf population	Wolves killed*cattle, wolves killed*sheep, wolves killed*wolf population, cattle*wolf population	557.22	−537.216
48	Wolves killed + cattle + sheep + wolf population	Wolves killed*cattle, wolves killed*sheep, wolves killed*wolf population, sheep*wolf population	552.26	−532.258
49	Wolves killed + cattle + sheep + wolf population	Wolves killed*wolf population, cattle*sheep, cattle*wolf population, sheep*wolf population	553.62	−533.622
50	Wolves killed + cattle + sheep + wolf population	Wolves killed*cattle, wolves killed*sheep, wolves killed*wolf population, cattle*sheep, cattle*wolf population	553.53	−531.534
51	Wolves killed + cattle + sheep + wolf population	Wolves killed*cattle, wolves killed*sheep, wolves killed*wolf population, cattle*sheep, sheep*wolf population	551.27	−529.273
52	Wolves killed + cattle + sheep + wolf population	Wolves killed*cattle, wolves killed*sheep, wolves killed*wolf population, cattle*sheep, cattle*wolf population, sheep*wolf population	553.20	−529.200
53	Wolves killed + cattle + sheep + wolf population	Wolves killed*cattle, wolves killed*sheep, wolves killed*wolf population, cattle*sheep, cattle*wolf population, sheep*wolf population, Wolves killed*cattle*wolf population, Wolves killed*cattle*wolf population, Wolves killed*sheep*wolf population, Cattle*sheep*wolf population, Wolves killed*cattle*sheep*wolf population	544.05	−510.052

**Table 4:** Summary of best following year sheep depredated models.

Dependent Variable	Independent Variable	Estimated Coefficients	Standard Error	VIF	Z value	p-value	AIC
**Sheep Depredated**	Wolves killed	0.03883	0.01751	3.26	2.218	0.026	544.05
	Minimum wolf population	0.05539	0.01720	4.29	3.220	0.001	
	Cattle	3.058×10^−5^	6.481×10^−6^	1.67	4.718	<0.001	
	Sheep	2.077×10^−4^	6.255×10^−5^	2.56	3.320	0.001	
	Wolves killed*wolf population	−5.116×10^−4^	2.703×10^−4^		−1.893	0.058	
	Wolves killed*cattle	−4.932×10^−7^	2.707×10^−7^		−1.822	0.068	
	Wolf population*cattle	−1.159×10^−7^	3.657×10^−8^		−3.170	0.001	
	Wolves killed*sheep	−3.712×10^−6^	2.097×10^−6^		−1.770	0.077	
	Wolf population*sheep	−6.827×10^−7^	2.610×10^−7^		−2.616	0.009	
	Cattle*sheep	−3.408×10^−10^	7.444×10^−11^		−4.578	<0.001	
	Wolves killed*wolf population*cattle	6.532×10^−10^	5.772×10^−10^		1.132	0.258	
	Wolves killed*wolf population*sheep	4.819×10^−9^	4.247×10^−9^		1.135	0.256	
	Wolves killed*cattle*sheep	3.682×10^−12^	3.216×10^−12^		1.145	0.252	
	Wolf population*cattle*sheep	1.534×10^−12^	5.635×10^−13^		2.722	0.006	
	Wolves killed*wolf population*cattle*sheep	−4.336×10^−15^	8.803×10^−15^		−0.493	0.622	

**Figure 4:**
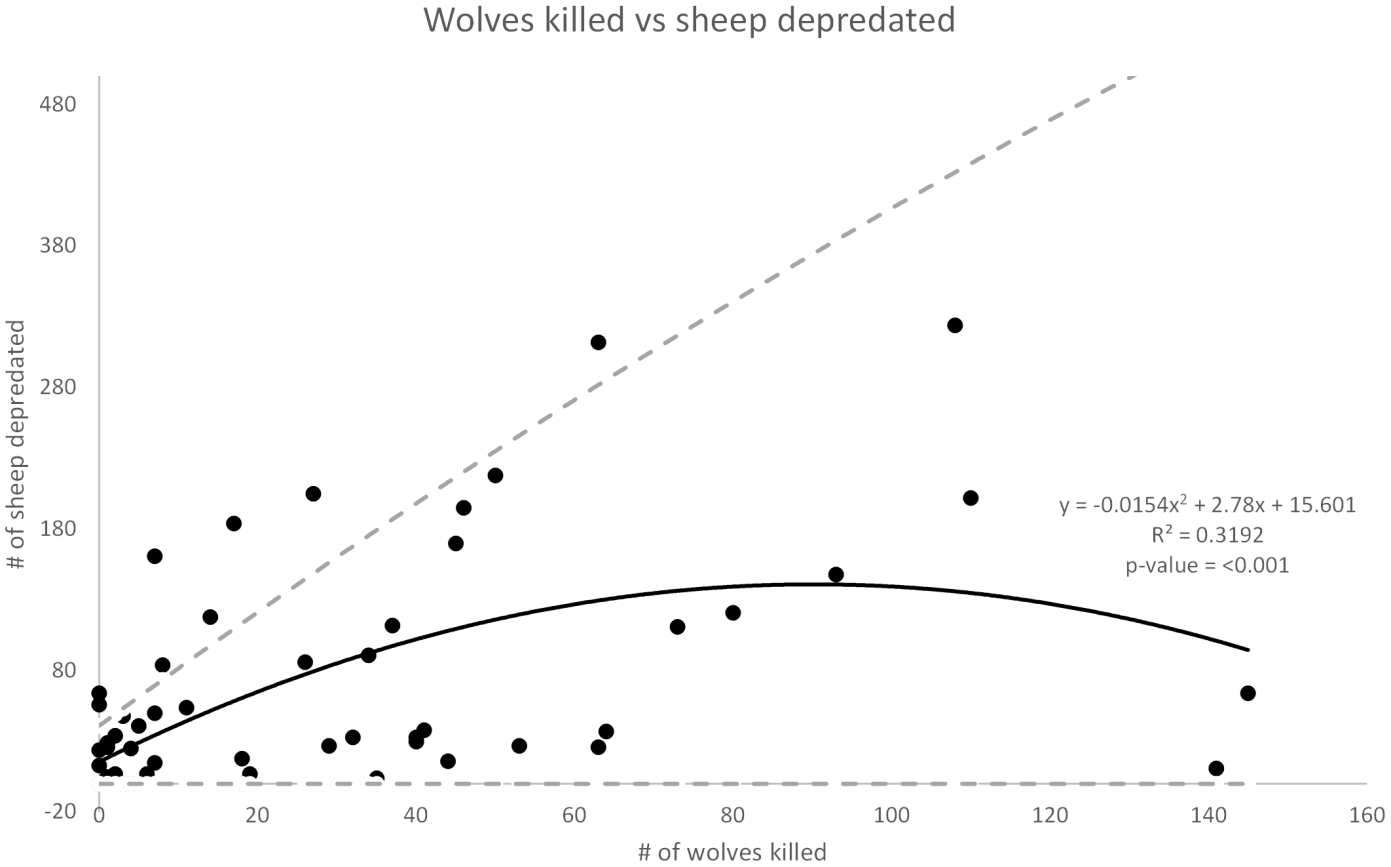
Wolves killed vs sheep depredated. Number of wolves killed through control methods the previous year versus the number of sheep depredated the following year. The dashed lines show the upper and lower limits of the 95% confidence interval for the best fit line.

**Figure 5:**
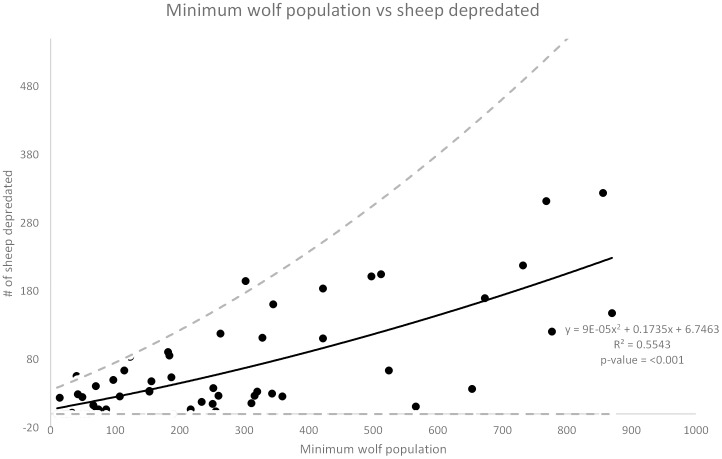
Minimum wolf population vs sheep depredated. Minimum year end wolf population the previous year versus the number of sheep depredated the following year. The dashed lines show the upper and lower limits of the 95% confidence interval for the best fit line.

**Figure 6:**
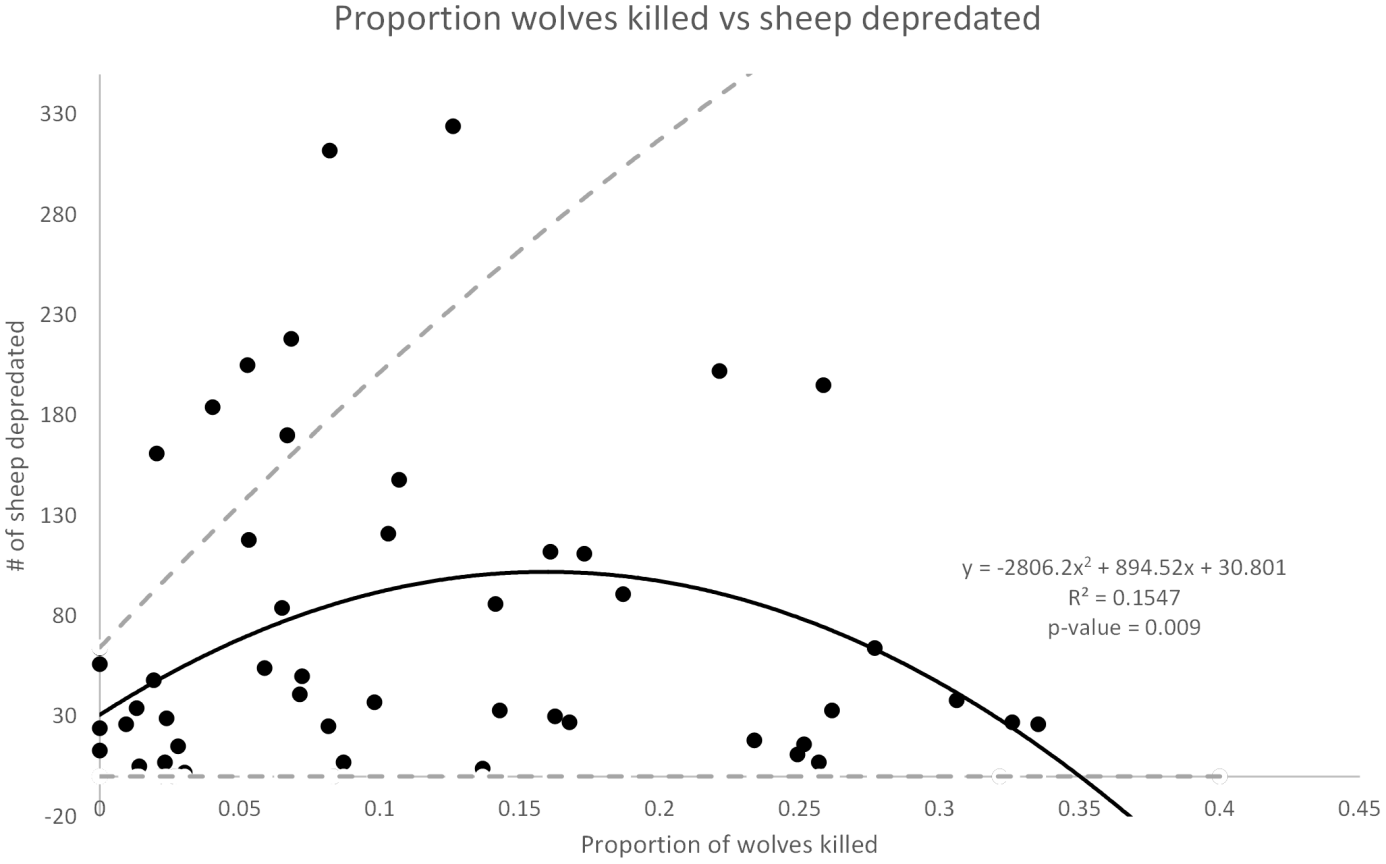
Proportion of wolves controlled versus the number of sheep depredated. Proportions of wolves killed through control methods the previous year versus the number of sheep depredated the following year. The dashed lines show the upper and lower limits of the 95% confidence interval for the best fit line.

Both of the main effects and one interaction effect were significant in this model. Once again, the number of wolves killed was positively related to the number of sheep depredated the following year (rate ratio  = 1.04, *z* = 2.218, *P* = 0.026) (Figure 4). For each additional wolf killed the estimated mean number of sheep being depredated the following year increased by 4%. The minimum wolf population was also positively related to the number of sheep depredated the following year (rate ratio  = 1.06, *z* = 3.220, *P* = 0.001) (Figure 5). For each additional wolf on the landscape the estimated mean number of sheep being depredated the following year increased by 6%. The number of cattle and sheep were found to be positively related to the number of sheep depredated but the coefficient was negligible (rate ratios  = 1.00 and 1.00, *z* = 4.718 and 3.320, *P* = <0.001 and 0.001) which results in an increase of sheep depredated the following year by 1.00 or less than 1%. However, as with cattle, there was an important 2-way negative interaction. Sheep depredations increased with increasing wolf mortality rate up until about 25%, then depredations began to decline after mortality exceeded 25% (Figure 6).

## Discussion

Our results do not support the “remedial control” hypothesis of predator mortality on livestock depredations the following year. However, lethal control of wolves appears to be related to increased depredations in a larger area the following year. Our results are supported by the findings of Harper et al. (2008) in Minnesota where they found that across the state (large scale) none of their correlations supported the hypothesis that killing a high number of wolves reduced the following year's depredations. Harper et al also found that trapping and not catching wolves decreased depredations more than no trapping at all, suggesting that a mere increase in human activity at depredation sites reduced further depredations by those wolves in their study area. By contrast, Bjorge and Gunson (1985) found reducing the population from 40 to 3 wolves in 2 years in Alberta (a 10 fold reduction to near extirpation) resulted in a decline of livestock depredations for two years - followed by subsequent recolonization and increased depredations thereafter. Tompa (1983) also found that lethal control prevented conflict for more than a year in some areas of British Columbia. It should be noted that these 2 studies examined wolf control and livestock depredations at a fine scale (grazing allotment or wolf pack territory or management zone). They did not examine wolf control and livestock depredations at a larger scale (wolf occupied areas) as was done by Harper et al. (2008) and us (this study). It appears that wolf control is associated with reduced depredations at the local wolf pack scale but increased depredations at the larger wolf population scale. This appears consistent with Treves et al. (2005) prediction that the removal of carnivores generally only achieves a temporary reduction in livestock depredations locally when immigrants can rapidly fill the vacancies.

There were several different factors that influenced the number of livestock depredated the following year by wolves. In order of importance, based on the values of the rate ratios, these include: the number of wolves removed through control methods, the number of breeding pairs, the minimum wolf population, and the number of livestock on the landscape. Consistent with expectations, each additional breeding pair on the landscape increased the expected mean number of cattle depredated by 8 to 9% and each additional wolf on the landscape increased the expected mean number of sheep depredated by 6%. Cattle were most affected by breeding pairs and sheep by wolves – perhaps because it takes more than one wolf (a pack) to kill a relatively larger cow and only one wolf to kill a smaller sheep. However, contrary to the “remedial control” hypothesis, each additional wolf killed increased the expected mean number of livestock depredated by 5–6% for cattle and 4% for sheep. It appears that lethal wolf control to reduce the number of livestock depredated is associated with increased, not decreased, depredations the following year, on a large scale – at least until wolf mortality exceeds 25%. Why 25%? The observed mean intrinsic growth rate of wolves in Idaho, Wyoming, and Montana is about 25% [21]. Therefore, once anthropogenic mortality exceeds 25%, the numbers of breeding pairs and wolves must decline – resulting in fewer livestock depredations.

Below 25% mortality, lethal control may increase breeding pairs and wolves through social disruption and compensatory, density dependent effects. For example, wolf control efforts occur year round and often peak during grazing season in areas with livestock depredations [22], [23]. However, if control takes place during the breeding season and a member of the breeding pair is removed it may lead to pack instability and increased breeding pairs [24], [10]. Furthermore, loss of a breeder in a pack during or near breeding season can result in dissolution of territorial social groups, smaller pack sizes and compensatory density dependent effects – such as increased per-capita reproduction [11], [25], [26]. Culling of wolves may also cause frequent breeder turnover [11] and related social disruption – which can result in reduced effective prey use (through loss of knowledge of prey sources and ability to subdue prey) which may also result in increased livestock depredations [27], [28]. All of these effects could potentially result in increased livestock depredations.

We would expect to see increased depredations, wolves killed, and breeding pairs as the wolf population grows and recolonizes the area - but our data suggest that lethal control exacerbates these increases. The secondary effects of time, wolf population growth rate, wolf occupied area, and wolf population size on depredations were already subsumed in the primary main effect terms of breeding pairs (cattle) and wolves (sheep), so those secondary effects cannot account for the positive effects of wolf kills on depredations. We do not yet know the exact mechanism of how increased wolf mortality up to ≤25% results in increased livestock depredations, but we do know that increased mortality is associated with compensatory increased breeding pairs, compensatory numbers of wolves, and depredations [24], [10], [27], [28], [11], [26]. Further research is needed to determine the exact causal mechanism(s). Annual mortality in excess of 25% will reduce future depredations, but that mortality rate is unsustainable and cannot be carried out indefinitely if federal relisting of wolves is to be avoided. Furthermore, a 5% (sheep) and 5% (cattle) kill rate of wolves yields the same number of cattle and sheep depredations as a 35% (cattle) and 30% (sheep) kill rate (Figures 3 & 6), but the 30% or 35% rate is unsustainable for wolf population persistence and the 5% rate is not. The worst possible case appears to be a high mortality rate at about 20–25%, since this corresponds to a “standing wave” of the highest livestock depredations. Further research is needed to test if this high level of anthropogenic wolf mortality (25%) is associated with high levels of predation on natural prey such as deer and elk.

Further research is also needed to account for the limitations of our data set. The scale of our analysis was large (wolf occupied areas in each state in each year) and the scale of some other studies were small (wolf packs). Simultaneous, multi-scale analysis (individual wolf packs, wolf management zones, and wolf occupied areas) may yield further insights.

Although lethal control is sometimes a necessary management tool in the near-term, we suggest that managers also consider testing non-lethal methods of wolf control [29] because these methods might not be associated with increased depredations in the long-term.

## Supporting Information

Figure S1 **Proportion of wolves harvested vs cattle depredated.** Proportion of wolves harvested the previous year in each state (Montana, Idaho and Wyoming) versus the number of cattle depredated the following year.
(TIF)

Figure S2 **Proportion of wolves harvested vs sheep depredated.** Proportion of wolves harvested the previous year in each state (Montana, Idaho and Wyoming) versus the number of sheep depredated the following year.
(TIF)

Table S1 **Data by state, 1987–2012.** Data for all variables used in the analysis grouped by state from 1987–2012.
(DOCX)

Table S2 **Pearson correlation matrix.** Pearson correlation matrix for independent variables: cattle, sheep, minimum wolf population, wolves harvested and number of breeding pairs.
(DOCX)
