# Supplementary figures and images for: Effects of Wolf Mortality on Livestock Depredations
Source: PLoS One. 2014 Dec 3;9(12):e113505. doi: 10.1371/journal.pone.0113505 (PMC4254458; doi:10.1371/journal.pone.0113505)

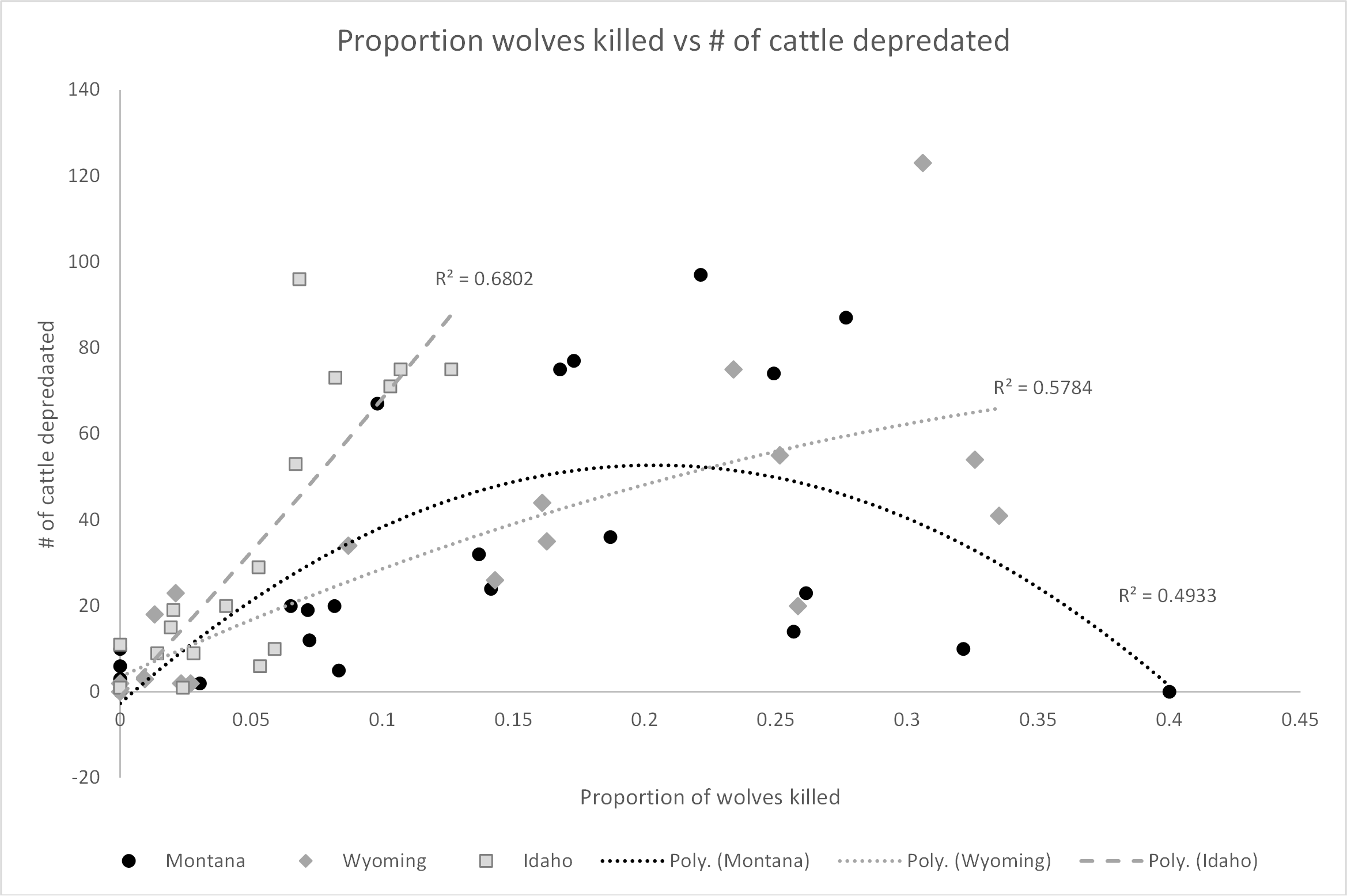

Supplement: Figure S1 — Proportion of wolves harvested vs cattle depredated. Proportion of wolves harvested the previous year in each state (Montana, Idaho and Wyoming) versus the number of cattle depredated the following year. (TIF) [file pone.0113505.s001.tif]

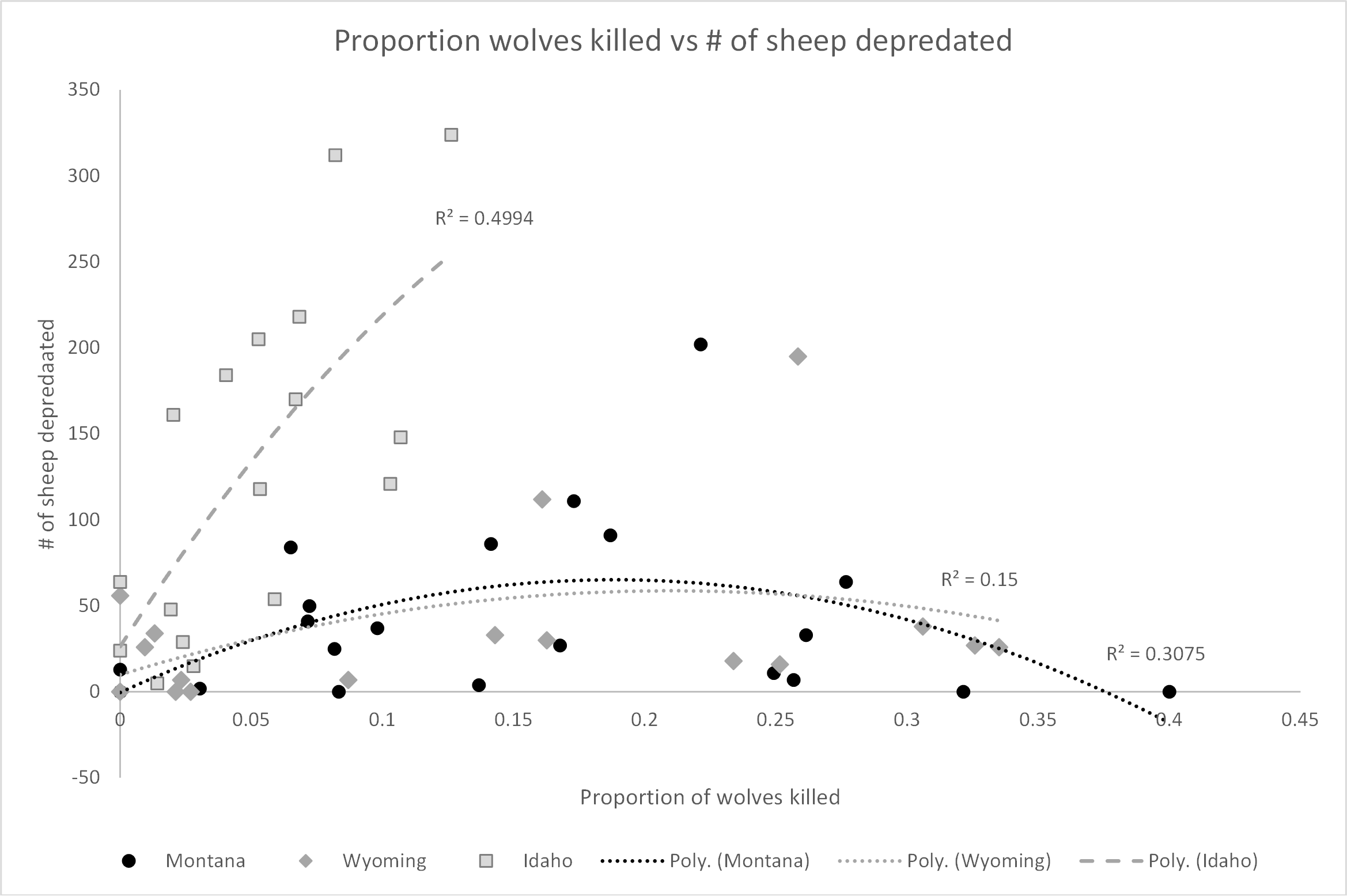

Supplement: Figure S2 — Proportion of wolves harvested vs sheep depredated. Proportion of wolves harvested the previous year in each state (Montana, Idaho and Wyoming) versus the number of sheep depredated the following year. (TIF) [file pone.0113505.s002.tif]
