# Supplementary material for: Effects of Wolf Mortality on Livestock Depredations
Source: PLoS One. 2014 Dec 3;9(12):e113505. doi: 10.1371/journal.pone.0113505 (PMC4254458; doi:10.1371/journal.pone.0113505)
Supplement: Table S1 — Data by state, 1987–2012. Data for all variables used in the analysis grouped by state from 1987–2012. (DOCX) [file pone.0113505.s003.docx]

| Year | 87 | 88 | 89 | 90 | 91 | 92 | 93 | 94 | 95 | 96 | 97 | 98 | 99 | 00 | 01 | 02 | 03 | 04 | 05 | 06 | 07 | 08 | 09 | 10 | 11 | 12 |
| --- | --- | --- | --- | --- | --- | --- | --- | --- | --- | --- | --- | --- | --- | --- | --- | --- | --- | --- | --- | --- | --- | --- | --- | --- | --- | --- |
| Montana |  |  |  |  |  |  |  |  |  |  |  |  |  |  |  |  |  |  |  |  |  |  |  |  |  |  |
| Cattle depredated | 6 | 0 | 3 | 5 | 2 | 1 | 0 | 6 | 3 | 10 | 19 | 10 | 20 | 14 | 12 | 20 | 24 | 36 | 23 | 32 | 75 | 77 | 97 | 87 | 74 | 67 |
| Sheep depredated | 10 | 0 | 0 | 0 | 2 | 0 | 0 | 0 | 0 | 13 | 41 | 0 | 25 | 7 | 50 | 84 | 86 | 91 | 33 | 4 | 27 | 111 | 202 | 64 | 11 | 37 |
| Wolf population | 10 | 14 | 12 | 33 | 29 | 41 | 55 | 48 | 66 | 70 | 56 | 49 | 74 | 97 | 123 | 183 | 182 | 152 | 256 | 316 | 422 | 497 | 524 | 566 | 653 | 625 |
| Wolves killed | 4 | 0 | 1 | 1 | 0 | 0 | 0 | 0 | 0 | 5 | 18 | 4 | 19 | 7 | 8 | 26 | 34 | 40 | 35 | 53 | 73 | 110 | 145 | 141 | 64 | 108 |
| Breeding pairs | 2 | 1 | 1 | 3 | 2 | 4 | 4 | 5 | 6 | 7 | 5 | 5 | 7 | 8 | 7 | 17 | 10 | 15 | 19 | 21 | 39 | 34 | 37 | 35 | 39 | 37 |
| Number of cattle^A^* | 9736 | 9324 | 9190 | 8345 | 8733 | 9428 | 9290 | 9304 | 9813 | 10009 | 9821 | 9346 | 9343 | 9287 | 9189 | 8855 | 8443 | 8130 | 8033 | 8220 | 8036 | 8562 | 8479 | 8327 | 8180 |  |
| Number of Sheep^B^* | 1478 | 1554 | 1572 | 1666 | 1639 | 1608 | 1385 | 1305 | 1195 | 1138 | 1096 | 1067 | 981 | 937 | 956 | 932 | 784 | 763 | 715 | 717 | 735 | 738 | 668 | 650 | 626 |  |
| Wyoming |  |  |  |  |  |  |  |  |  |  |  |  |  |  |  |  |  |  |  |  |  |  |  |  |  |  |
| Cattle depredated |  |  |  |  |  |  |  |  | 0 | 0 | 2 | 2 | 2 | 3 | 18 | 23 | 34 | 75 | 54 | 123 | 55 | 41 | 20 | 26 | 35 | 44 |
| Sheep depredated |  |  |  |  |  |  |  |  | 0 | 0 | 56 | 7 | 0 | 25 | 34 | 0 | 7 | 18 | 27 | 38 | 16 | 26 | 195 | 33 | 30 | 112 |
| Wolf population |  |  |  |  |  |  |  |  | 21 | 40 | 86 | 112 | 107 | 153 | 189 | 217 | 234 | 272 | 252 | 311 | 359 | 302 | 320 | 343 | 328 | 277 |
| Wolves killed |  |  |  |  |  |  |  |  | 0 | 0 | 2 | 3 | 1 | 2 | 4 | 6 | 18 | 29 | 41 | 44 | 63 | 46 | 32 | 40 | 37 | 43 |
| Breeding pairs |  |  |  |  |  |  |  |  | 2 | 4 | 9 | 6 | 7 | 12 | 13 | 18 | 16 | 25 | 16 | 25 | 25 | 22 | 27 | 27 | 27 | 21 |
| Number of cattle^A^* |  |  |  |  |  |  |  |  | 3310 | 3410 | 3510 | 3620 | 3420 | 3320 | 3270 | 2900 | 2630 | 2680 | 2620 | 2690 | 2670 | 2712 | 2781 | 2725 | 2684 |  |
| Number of Sheep^B^* |  |  |  |  |  |  |  |  | 790 | 780 | 740 | 720 | 700 | 770 | 690 | 660 | 660 | 1114 | 1120 | 1049 | 1025 | 816 | 804 | 722 | 700 |  |
| Idaho |  |  |  |  |  |  |  |  |  |  |  |  |  |  |  |  |  |  |  |  |  |  |  |  |  |  |
| Cattle depredated |  |  |  |  |  |  |  |  | 0 | 1 | 1 | 9 | 11 | 15 | 10 | 9 | 6 | 19 | 20 | 29 | 53 | 96 | 75 | 75 | 71 | 73 |
| Sheep depredated |  |  |  |  |  |  |  |  | 0 | 24 | 29 | 5 | 64 | 48 | 54 | 15 | 118 | 161 | 184 | 205 | 170 | 218 | 324 | 148 | 121 | 312 |
| Wolf population |  |  |  |  |  |  |  |  | 14 | 42 | 71 | 114 | 156 | 187 | 251 | 263 | 345 | 422 | 512 | 673 | 732 | 856 | 870 | 777 | 768 | 683 |
| Wolves killed |  |  |  |  |  |  |  |  | 0 | 1 | 1 | 0 | 3 | 11 | 7 | 14 | 7 | 17 | 27 | 45 | 50 | 108 | 93 | 78 | 63 | 73 |
| Breeding pairs |  |  |  |  |  |  |  |  |  | 3 | 6 | 10 | 10 | 10 | 14 | 13 | 28 | 26 | 36 | 41 | 43 | 39 | 49 | 46 | 40 | 35 |
| Number of cattle^A^* |  |  |  |  |  |  |  |  | 4893 | 4775 | 4704 | 4785 | 4859 | 4915 | 4810 | 4655 | 4220 | 4092 | 4068 | 3991 | 4074 | 3657 | 3502 | 3578 | 3686 |  |
| Number of Sheep^B^* |  |  |  |  |  |  |  |  | 872 | 796 | 841 | 857 | 799 | 810 | 854 | 892 | 818 | 829 | 821 | 766 | 309 | 267 | 234.12 | 245.27 | 257 |  |

Table S1. Data by state, 1987-2012

^A^Multiply by 100

^B^Multiply by 100

*since we used livestock counts from previous year to determine effect on depredations following year the number of livestock was not obtained for 2012 as the last year for depredations was 2012
