# Supplementary material for: Effects of Wolf Mortality on Livestock Depredations
Source: PLoS One. 2014 Dec 3;9(12):e113505. doi: 10.1371/journal.pone.0113505 (PMC4254458; doi:10.1371/journal.pone.0113505)
Supplement: Table S2 — Pearson correlation matrix. Pearson correlation matrix for independent variables: cattle, sheep, minimum wolf population, wolves harvested and number of breeding pairs. (DOCX) [file pone.0113505.s004.docx]

**Table S1. Pearson correlation matrix**

|  | Cattle | Sheep | Minimum wolf population | Wolves harvested | Number of breeding pairs |
| --- | --- | --- | --- | --- | --- |
| Cattle | 1.000 |  |  |  |  |
| Sheep | 0.536 | 1.000 |  |  |  |
| Minimum wolf population | -0.348 | -0.719 | 1.000 |  |  |
| Wolves harvested | -0.045 | -0.544 | 0.797 | 1.000 |  |
| Number of breeding pairs | -0.346 | -0.714 | 0.955 | 0.834 | 1.000 |
